# Bacterial Extracellular Vesicles in Gastrointestinal Tract Cancer: An Unexplored Territory

**DOI:** 10.3390/cancers13215450

**Published:** 2021-10-29

**Authors:** Sajeen Bahadur Amatya, Sonja Salmi, Veera Kainulainen, Peeter Karihtala, Justus Reunanen

**Affiliations:** 1 Biocenter Oulu & Cancer and Translational Medicine Research Unit, University of Oulu, 90014 Oulu, Finland; Sajeen.Amatya@oulu.fi (S.B.A.); Sonja.Salmi@oulu.fi (S.S.); 2 Human Microbiome Research Program Unit, Faculty of Medicine, University of Helsinki, 00290 Helsinki, Finland; veera.kainulainen@helsinki.fi; 3 Helsinki University Hospital Comprehensive Cancer Center, University of Helsinki, 00290 Helsinki, Finland; peeter.karihtala@hus.fi

**Keywords:** gastrointestinal tract, microbiota, extracellular vesicles, cancer

## Abstract

**Simple Summary:**

Microbial dysbiosis has been credited as one of the contributing factors to the development and progression of gastrointestinal tract cancer. The altered microbiota influences carcinogenesis through the induction of instability and damage to genetic material, modulation of host metabolic and inflammatory pathways, production of carcinogenic metabolites, and suppression of host antitumor response. These microbes secrete extracellular vesicles that are possibly carrying carcinogenic bioactive metabolites within their cargo. Studies have illustrated the ability of bacterial extracellular vesicles to cross the intestinal epithelial barrier and selectively accumulate near intestinal tumor cells. The purpose of this systemic review was to highlight the possible role of gut bacterial vesicles in the development, progression, and pathogenesis of gastrointestinal tract cancer and their possible involvement in the modulation of the tumor microenvironment. An infinitesimal amount of research has been carried out on the impact of bacterial extracellular vesicles on oncogenesis and tumor progression. This review aimed to encourage more investigations on this subject.

**Abstract:**

Bacterial extracellular vesicles are membrane-enclosed, lipid bi-layer nanostructures that carry different classes of biomolecules, such as nucleic acids, lipids, proteins, and diverse types of small molecular metabolites, as their cargo. Almost all of the bacteria in the gut secrete extracellular vesicles to assist them in competition, survival, material exchange, host immune modulation, infection, and invasion. The role of gut microbiota in the development, progression, and pathogenesis of gastrointestinal tract (GIT) cancer has been well documented. However, the possible involvement of bacterial extracellular vesicles (bEVs) in GIT cancer pathophysiology has not been given due attention. Studies have illustrated the ability of bEVs to cross physiological barriers, selectively accumulate near tumor cells, and possibly alter the tumor microenvironment (TME). A systematic search of original published works related to bacterial extracellular vesicles on gastrointestinal cancer was performed for this review. The current systemic review outlines the possible impact of gut microbiota derived bEVs in GIT cancer in light of present-day understanding. The necessity of using advanced sequencing technologies, such as genetic, proteomic, and metabolomic investigation methodologies, to facilitate an understanding of the interrelationship between cancer-associated bacterial vesicles and gastrointestinal cancer is also emphasized. We further discuss the clinical and pharmaceutical potential of bEVs, along with future efforts needed to understand the mechanism of interaction of bEVs in GIT cancer pathogenesis.

## 1. Introduction

The human gastrointestinal system is one of the most complex known microbial systems; it is colonized by trillions of microorganisms, including bacteria, archaea, fungi, and viruses [1,2], with bacteria being the largest group [3]. Studies have indicated that microbiota influence the development and progression of cancer by modulating the tumor microenvironment (TME) [4,5,6,7]. The alteration in the gastrointestinal tract (GIT) microbiota, with an increase in pathogenic bacteria concomitant with a decrease in beneficial bacteria, is observed during the development of gastrointestinal cancers [2,8]. Gut microorganisms maintain host homeostasis and immunity, referred to as eubiosis [9], by utilizing different metabolic and immunomodulatory properties to sustain a balanced host health status. Alterations in microbial community in GIT may lead to a loss of ability to maintain homeostatic conditions, which contributes to cancer pathogenesis and progression [9,10]. Studies suggest that dysbiosis of the gut microbiome also alters its metabolic products, influencing host metabolic and inflammatory pathways, thereby disturbing homeostasis and paving the way to carcinogenesis [11]. Microbial dysbiosis has also been attributed to reduced responses to anticancer therapies due to the ability of certain microbes to metabolize drugs and influence immune responses within the TME [12]. Substantial evidence indicates that the carcinogenic effects of microbiota can be transferred to healthy mice by fecal microbiota transfer (FMT) from mice or human patients suffering from GIT cancer [13].

Extracellular vesicles (EVs) have been known to be produced by almost all forms of life including eukaryotes and prokaryotes [14,15]. EVs have also been found in different biofluids such as saliva [16], serum [17], plasma [17], amniotic fluid [16], breast milk [18], and urine [19]. Bacterial extracellular vesicles (bEVs) are membrane-enclosed lipid bi-layer structures secreted by almost all known bacteria. Bacteria secrete EVs that range from 20 to 400 nm in size [20,21] and contain different classes of biomolecules such as nucleic acids [22,23,24], lipids [25,26], proteins [27,28,29], and diverse types of small molecular metabolites [30,31]. bEVs display multiple bacterial cell surface components, such as lipopolysaccharide (LPS) [32,33], peptidoglycan (PG) [34,35], outer membrane proteins [36], enzymes [37,38], and toxins [39,40], many of which belong to the microbe-associated molecular patterns (MAMPs) or pathogen-associated molecular patterns (PAMPs) [37,41]. Thus, many bEVs have the ability to stimulate different pattern recognition receptors (PRRs) such as Toll-like receptors [42,43] and to activate different signaling pathways related to cytokines [41,44], chemokine [45], and inflammatory responses [41,42] in the host cell. These inflammatory substances have been observed to play an active role in tumor pathogenesis [46,47,48]. In addition, certain bEVs have been shown to selectively bind to the host cell surfaces [49,50]. bEVs are believed to facilitate cell-to-cell communication among microbes and also between host and microbe. They regulate recipient cells, for instance, via horizontal transfer of genetic material [51,52,53,54], delivery of toxins and virulence factors [38,55], and antimicrobial substances [56,57]. bEVs serve as efficient delivery vehicles for different microbial bioactive substances and genetic materials, since within the vesicles, they are protected from degradation by RNases, DNases, and other host enzymatic and immune activities by the lipid envelope [22,23,58].

The possibility of changes in the gene expression of cancer cells due to the horizontal transfer of exogenous genetic material from the gut microbiome has previously been speculated [59,60]. Bacterial genetic material has been found to be integrated in the DNA of human cancer samples much more frequently than in DNA samples from healthy individuals [60], raising the possibility of bacterial regulation of host cells, either by direct integration of bacterial genetic material or by epigenetic alteration, through the production of pro-carcinogenic proteins and enzymes [61]. It can be speculated that the migration of genetic material or pro-carcinogenic bacterial material to the cancer site is feasible if we consider the possibility of the involvement of bacterial vesicles in their transport, as these bacterial foreign materials would be safe as they would easily evade the host immune mechanism, being enclosed inside the membrane envelope. Studies have indicated that the packaging of cargo into bEVs is not random but rather a well-organized selective process, whereby bacteria transfer their nucleic acid [62], proteins [36,55], virulence factors, toxins, and other metabolites [36,43,63] into secreted vesicles. Thus, we can presume that this selective packaging gives bacterial vesicles an evolutionary advantage whereby the individual bacterial species can influence other bacteria and host cells according to their needs, e.g., for a symbiotic relationship [57,64] or for the destruction of competing bacteria [65]. Similarly, bacteria may also influence the host cells by modulating the host genetic or physiological environment, leading to the initiation of a diseased condition.

EVs secreted by tumor cells have been known to be involved in the development and progression of tumors by facilitating interaction between tumor and stroma [66,67], protecting a tumor from the host immune system [68], initiation of angiogenesis [69,70], invasion of tumor extracellular matrix [71,72], and even the metastasis [73,74] of tumor cells to new locations. A large number of studies related to the effects of tumor cell-derived vesicles on tumor pathogenesis, development, progression, and metastasis have been carried out by many research groups [75,76], but the role of bacterial vesicle counterparts has been largely undermined. Limited studies performed in that direction indicate the possible involvement of bEVs in TME. These investigations indicate that bEVs showed increased affinity to cancer cells by being accumulated in the surrounding space as well as being easily transported inside the cancer cells [20,49,77]. Further studies are required to reveal the reasons for the targeted affinity of these vesicles to tumor cells, mechanisms of entry, and their roles in the modulation of a cell’s genetic and cytosolic environment in relation to cancer pathogenesis.

Similarly, there seems to be a mutual exchange of metabolites between cancer cells and microorganisms aided by their respective vesicles [78]. Contrary to the involvement of bEVs in cancer pathogenesis, different kinds of integrins, proteins, and keratins that are specific to EVs derived from colorectal cancer (CRC) have been detected in altered microbial populations of GIT, which offers evidence of the modulation of gut microorganisms by cancer cells. In their review, Barteneva et al. (2017) [15] tabulated the matching protein sequences in GIT microorganisms with data from proteomic analysis of CRC-derived EVs.

Despite an enormous interest in the role of EVs and a plethora of separation methods available for the isolation and separation of EVs, there is no consensus on reliable isolation and analysis protocols [79,80]. Most of the available EV isolation and analysis protocols are focused on human EVs [79,81,82,83]. Few articles on bEV isolation mention the methodological challenges in separating bEVs from host-derived EVs as one of the main reasons for bEVs being less thoroughly investigated in relation to human health [80,84]. High-throughput methods of “omics” have become a standard research tool for the study of microorganisms in recent years [85]. The -omics technology has made it possible to identify the viable but unculturable microorganisms in the gut [86]. The advances in -omics technologies have revolutionized the understanding of the microbial community, its composition, and its activity as a whole [87]. The use of these advancements has been very limited in the study of bEVs in relation to GIT cancer. Implementation of proper purification techniques is essential in order to obtain reliable -omics data and also to identify EV-specific functions and biomarkers [79]. Conversely, the knowledge gained by different -omics studies may be implemented to devise reliable guideline for the isolation, purification, and further study of bEVs.

This review discusses the probable role of bacterial secreted vesicles in the development, progression, and pathogenies of cancers affecting the GIT in the light of our current understanding. The effects of cancer-associated gut microorganisms on the pathology of intestinal cancers are well documented, but their interrelationships with bEVs are only starting to be investigated. A large number of studies regarding host tumor tissue secreted membrane vesicles have also been carried out, but those of bEVs are largely missing. The emphasis of this review is to highlight the necessity of using the latest gene sequencing methods along with proteomics and metabolomics in order to increase the scope of knowledge regarding the inter-relationship between bEVs and GIT cancer.

## 2. Method

**Figure 1:**
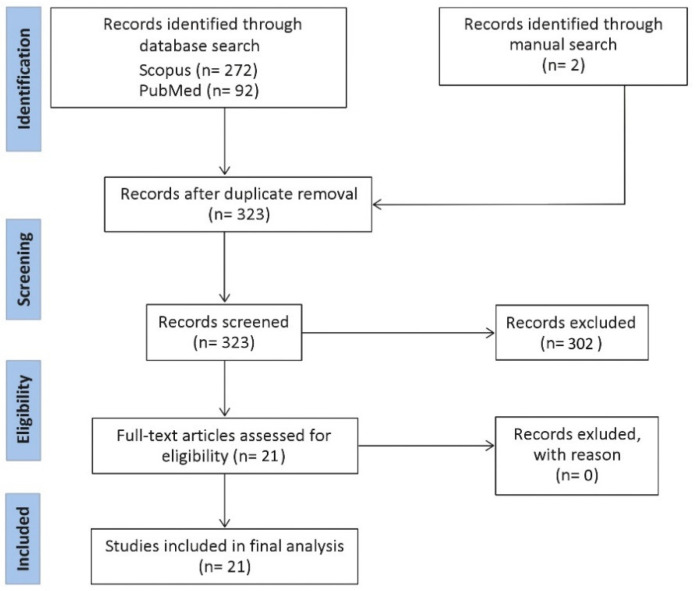
Flow diagram representing a summary of the methodology for this review.

A Scopus and PubMed search were conducted. The query combined four separate search items: (i) “bacteria”, including bacteria, microbiome; (ii) extracellular vesicle, including outer membrane vesicle, bEV, exosome, microvesicle; (iii) gastrointestinal tract, including oral, esophagus, gastric, stomach, gut, pancreas, liver, small intestine, large intestine, jejunum, duodenum, ileum, caecum, colon, colorectal, and rectum; (iv) cancer, including tumor, adenoma, carcinoma, malignant, neoplasm. No time interval was introduced. Only original studies written in English were considered. The retrieved records were collected into Covidence. All abstracts were critically assessed to select only those providing meaningful information related to the topic. Only studies related to bEVs on GIT cancers were included. A flow diagram with a summary of the methodology is provided in Figure 1.

## 3. GIT Cancer Statistics

Gastrointestinal cancer has high incidence, mortality, and morbidity rates according to the latest estimates of the Global Cancer Statistics 2020 (Table 1).

## 4. GIT Cancer-Associated Microbiota

A human host harbors about around 10^13^–10^14^ bacteria in his/her body, with the colon accommodating the highest number as reviewed in [7,13,90]. This large number of microbes contributes to a significant amount of genetic diversity in the human host, with the estimated number of genes contributed by intestinal microbiota being at least 100 times that of human genes as reviewed in [13,90]. Although these microorganisms are generally credited with the maintenance of homeostasis in the gastrointestinal environment, studies suggest that altered diversity and dysbiosis of gut bacteria lead to numerous gut-related diseases including GIT cancer [91,92,93]. Our microbiome plays a vital role in the maintenance of health, metabolism, and the immune system, and these components are also affected by a shift in the population of gut bacteria [64,94]. The association between gut bacteria and intestinal carcinogenesis was first suggested in 1974, when a carcinogenic substance injected into germ-free rats showed a lower incidence of GIT cancer than the colonized counterpart [13,95]. Since then, a large number of studies have broadened our understanding regarding the role of gut microorganisms in cancer pathogenesis [96,97,98].

A normal oral microbiome is composed of more than 600 different bacterial species. Alteration of the oral microbiome has been associated with oral precancerous lesions and oral carcinomas [99]. The microorganisms that have been strongly correlated with oral squamous cell carcinoma (OSCC) include *Porphyromonas gingivalis* [100] and *Capnocytophaga gingivalis* [101], and members of the genera *Streptococcus* [102], *Peptostreptococcus* [102], and *Prevotella* [101]. Other bacteria found in higher numbers in OSCC patients as compared with healthy individuals include *Fusobacterium nucleatum*, *Prevotella melaninogenica*, *Streptococcus mitis*, and members of the genera *Rothia*, *Gemella*, and *Lactobacillus* [103,104]. The most common bacteria associated with esophageal cancer are *Escherichia coli* [105], *Porphyromonas* *gingivalis* [106], *Fusobacterium nucleatum* [107], and members of *Lactobacillus* [108] and *Enterobacteriaceae* [109].

*Helicobacter pylori* has been listed as a major causative agent for gastric cancer [110]. Other bacteria common in gastric cancer are *Lactobacillus coleohominis* [111], *Klebsiella pneumoniae* [112], *Acinetobacter baumannii* [112], and members of the genera *Streptococcus* [92], *Veillonella*, *Prevotella* [92], *Fusobacterium* [113], *Lachnospiraceae* [114], *Leptotrichia* [115], and *Clostridium* [116].

Similarly, an increased prevalence of *Fusobacterium nucleatum* [117], *Escherichia coli* [118], *Streptococcus bovis* [119], *Streptococcus gallolyticus* [119], *Bacteroides fragilis* [6], species of *Dorea* [120] and *Porphyromonas* [120], and a diminished number of *Pseudomonas*, *Prevotella*, *Acinetobacter*, and *Catenibacterium* [121] are associated with CRC.

The majority of hepatic cancer cases are associated with the hepatitis B and C virus and an increased abundance of *Clostridium* [12], *Bacteroides* [122], and *Ruminococcaceae* [122].

*Helicobacter pylori* [123], *Pseudomonas aeruginosa* [124], and *Fusobacterium* [125] are associated with pancreatic cancer. 

Table 2 illustrates dysbiosis in GIT cancers.

FMT experiments, from tumor-bearing hosts to germ-free mice, have been reported to induce carcinogenesis in the recipient mice [149,150]. These studies illustrate the influence of cancer-associated bacteria in the initiation and progression of carcinogenesis [151,152]. Wong et al. (2017) [149] observed the promotion of tumorigenesis by the upregulation of proinflammatory genes and oncogenic factors as well as increased immune cell infiltration, when germ-free C57BL/6 mice were inoculated with feces from patients with CRC [149]. Li et al. (2019) [150] showed enhanced progression of intestinal adenoma in *Apc*^min/+^ mice via activation of the Wnt signaling pathway in the mice fed with gavage from CRC patients [150]. Similar results were obtained by Sobhania et al. (2019) [153] when fecal microbiota from CRC patients were transferred to germ-free mice, resulting in the induction of tumorigenesis via gene methylation [153]. Similarly, FMT experiments have also indicated that fecal microbiota transfers from drug-sensitive patients or healthy donors enhances the antitumor immune responses [154,155].

A large number of studies conducted over the past few decades have attributed the interaction between host cells and microorganisms as one of the major factors for the development and progression of cancer by modulating various host physiological processes [156,157,158]. The ability of cancer-associated intestinal bacteria to stimulate pro-tumor inflammation [46,159], the production of carcinogenic metabolites [159], the activation of carcinogenic signaling pathways [46], the induction of instability and damage to genetic material [160], the inhibition of apoptosis [12,161], the alteration of an antitumor immune response [162], among many other mechanisms, have been associated with intestinal cancer pathogenesis. Many gut microbiota produce metabolites that cause genetic instability and damage to DNA [160,163]; thus, the presence of such bacteria or their membrane vesicles can be directly linked to mutagenesis, resulting in cancer. The ability of microorganisms to influence cancer development and progression suggests a possible interaction between TME and gut microbiota.

## 5. Bacterial Extracellular Vesicles

**Figure 2:**
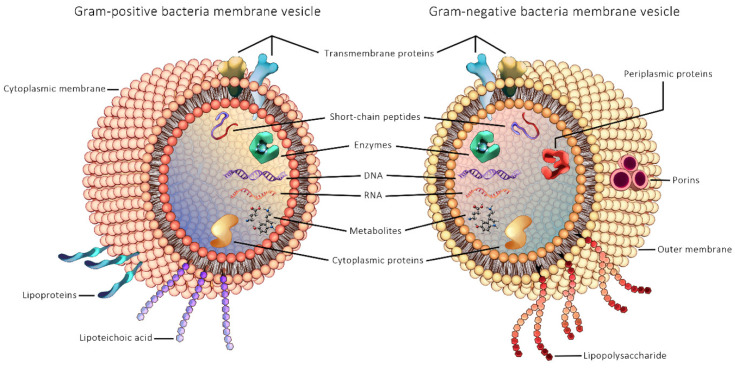
General structure of Gram-positive and Gram-negative bacterial extracellular vesicles.

Microbe-derived EVs have emerged as an important novel research topic in the context of understanding the role of gut microbial communities in human health and disease. The first study of bEVs reported secreted bEVs produced by the Gram-negative bacteria *Escherichia coli* in cultures in 1966 [164], while EVs from Gram-positive bacteria was first published only in 2009, from *Staphylococcus aureus* [165]. The reason for the delayed discovery of vesicles from Gram-positive bacteria has been attributed to the thick PG cell wall of Gram-positive bacteria that was assumed to act as a barrier to their release [165]. General structure of Gram-positive and Gram-negative bEVs are illustrated in Figure 2.

The EVs of Gram-negative bacteria are produced by controlled blebbing of the outer membrane and then released into the external environment; thus, they are called outer membrane vesicles (OMVs) as reviewed in [166]. The actual mechanism of OMV biogenesis is poorly understood, but several mechanistic models of their discharge have been proposed. Hoekstra et al. (1976) [167] suggested that the cleavage of the bond between the peptidoglycan layer and outer membrane leads to a bulging of OMVs followed by their detachment from the bacterial surface [167]. Another model suggests the accumulation of misfolded proteins or LPS or PG fragments leads to a depletion of crosslinks between PG and LPS and an increase in turgor pressure, and the release of OMVs finally occurs by the bulging of these vesicles through the bacterial surface [168,169,170]. Roier et al. (2016) [171] proposed the formation of OMVs due to the accumulation of phospholipids as a result of the deletion or decreased expression of *vacj* and *yrb* genes [171]. Recent studies suggested a new pathway leading to the formation of vesicles named as the outer-inner membrane vesicles (OIMVs) and explosive outer-membrane vesicles (EOMVs) based on explosive cell lysis triggered by the enzymatic action of endolysins [172,173,174,175]. Very little is known about the process of the creation of a membrane vesicle in Gram-positive bacteria. Membrane vesicle genesis in the Gram-positive bacteria *Bacillus subtilis* and *Staphylococcus aureus* were shown to be induced by the hydrolytic action of endolysins and autolysins, respectively, whereby cytoplasmic membrane materials protrude through the hole in the PG layer of the cell wall, resulting in the release of the vesicles [176,177].

Initially, membrane vesicle production was thought to be related to a stress response or an adaptive response to an adverse environment as a result of membrane instability [168,178]; later, findings suggested that the production of membrane vesicles is a well-regulated process with selective packaging of the components [178,179,180,181,182]. Proteomic analysis carried out on OMVs derived from *Escherichia coli* resulted in highly enriched outer membrane proteins, while inner membrane proteins were deficient, suggesting that the formation of a vesicle is not a random process [29]. Studies have demonstrated the selective incorporation of specific virulent protein gingipain in EVs produced by the human oral pathogen *Prophyromonas gingivalis* [36,183]. Similarly, proteomic analysis of *Helicobacter pylori*-derived vesicles conducted at different stages of growth revealed varying protein cargo, also reinforcing the notion of selective packaging [179].

bEVs carry a diversity of cargo compounds within them, including nucleic acids, proteins, and toxins, which help them in competition [165], survival [165], material exchange [165], host immune modulation [184,185], and infection [58]. Emerging evidence suggests that there exists a host–microbe interspecies communication between human and microorganisms, possibly aided by microbial EVs, which modulates certain human host functions [186,187]. The long-standing evolutionary connection between microorganisms and host may have enabled microorganisms to coexist in a close relationship with humans. Different studies have established that bEVs have evolved as a novel secretory mechanism employed by bacteria to deliver various cargo into the host cells without the need for the bacteria to have contact with the host cells [165,188,189,190]. *Escherichia coli* OMV have been found to fuse with lipid rafts in the host colonic epithelial cell [190]. Studies have demonstrated that bEVs fuse with lipid rafts in the plasma membrane to deliver multiple virulence factors directly to the host cytoplasm in a coordinated manner [189,190,191]. Koeppen and Hampton (2016) [24] demonstrated a pathogen–host interaction that reduces the innate immune response in the airway epithelial cells of mice and humans by a regulatory sRNA contained inside EVs secreted by *Pseudomonas aeruginosa* residing in infected cells [24]. bEVs were found to deliver LPS into the target cell cytosol, triggering caspase-11-dependent effector responses leading to innate immune responses during infection with Gram-negative bacteria such as *Escherichia coli* [192,193]. During infection, bEVs of bacteria, such as *Streptococcus pneumonia*, *Bacillus subtilis*, *Staphylococcus aureus*, *Helicobacter pylori*, and *Pseudomonas aeruginosa* [194,195], have been found to effectively alter the host cell environment, making it favorable for the bacteria to survive within the host cell [196]. As bEVs may contribute as vehicles that carry necessary cargo for this purpose, these EVs can be speculated to be a very efficient communication vehicle that carry information in forms of active signaling molecules between same and different species [189,197,198].

The isolation and purification of bEVs is a difficult process and often requires expensive equipment and complex purification protocols. Commonly used techniques for EV isolation are precipitation [169,199] and ultrafiltration [84,200]; the techniques used for EV purification are ultracentrifugation [80,201], density gradient fractionation [202,203], and immunoaffinity [81,84]. Work on bEV isolation protocols is still in its infancy. Patel et al. (2019) [204] demonstrated that the choice of isolation protocol can lead to a change in the yield, protein quantification, size distribution, and surface charge of EVs [204]; thus, isolation methods should be chosen carefully [205]. However, a detailed discussion of the isolation, purification, and characterization techniques of EVs is outside the scope of this review but has been extensively reported elsewhere as reviewed in [80,81,82,83,202,205,206,207,208].

The emerging role of bEVs in cancer and other physiological and pathological conditions has created a pressing need for an efficient labeling and tracking procedure to visualize and detect bEVs in target organs [209,210]. Non-invasive imaging modalities can provide an accurate understanding of the distribution and kinetics of bEVs in in vivo conditions [210]. Different labeling agents have been used for the study of EVs such as lipophilic tracer dyes [211,212], magnetic particles [213], radionuclides [214], and fluorescence [215,216]. The in vivo distribution, kinetics, dynamics, and fate of bEVs are largely unexplored. Most studies have focused on the study of EVs derived from different non-bacterial cells, and only a handful of studies have been conducted on bEVs. Fluorescent dyes, such as Rhodamine-R18, have been utilized to label OMVs from *Pseudomonas aeruginosa* [189] and *Aggregatibacter actinomycetemcomitans* [191] to study the fusion of OMVs with the host cell. Rhodamine-R18 only fluoresces upon fusion of OMVs to the host cell membrane and, thus, an increase in fluorescence can be interpreted as an increase in OMV fusion; this allows for visual confirmation and quantitation of OMV fusion with host cells [189,191]. Similarly, different dyes, such as 3,3′-dioctadecyloxacarbocyanine perchlorate (DiO), 1,1′-dioctadecyl-3,3,3′,3′-tetramethylindodicarbocyanine perchlorate (DiD) [217,218], fluorescein isothiocyanate (FITC) [190], have been used to investigate bEV–host cell interactions. The use of these labeling and tracking methods may also help to understand the interaction of these bEVs with host cells by injecting them into germ-free mice. This might eliminate the microbiota component from the equation and help us understand the impact of bEVs in cancer initiation and development in the absence of causative microorganisms.

## 6. Bacterial Extracellular Vesicles in the Tumor Microenvironment

The TME is composed of a complex ecosystem containing diverse types of cells, including cancer cells, cancer-associated fibroblasts, endothelial cells, adipocytes, mesenchymal stem cells, immune cells such as macrophages, T cells, B cells, and infiltrated cytokines, as well as non-cellular components, such as micro vesicles, and the extracellular matrix as reviewed in [219,220,221,222]. Studies have indicated that the interaction between tumor cells, the neighboring cells, and immune cells moderates tumor growth, progression, and metastasis as reviewed in [223,224]. Studies carried out on tumor cell-derived EVs have shown that they play a significant role in regulating key signaling pathways to modulate the TME and, thus, tumor progression. These studies even indicate that EVs derived from tumor stromal cells are capable of affecting the various properties of cancer cells, such as drug resistance, proliferation, and their ability to evade the immune mechanism, and may even take part in forming a new niche for metastatic cells in distant organs [225]. Though EVs derived from host cancer cells and cancer stroma cells have been extensively studied with respect to their role in tumor progression, drug resistance, angiogenesis, tumor proliferation, and metastasis as reviewed in [223,224], similar studies on bEVs have been largely overlooked.

Intestinal bEVs have been reported to enter the host circulatory system and have been found in the nearby organs [226,227]. Though the entry of bEVs into the host blood stream is generally attributed to conditions compromising the barrier integrity of intestinal epithelial cells, recent investigations reveal this phenomenon to be common in healthy individuals as well [80,228]. A mouse model study showed that bEVs isolated from the blood of mice contained bacterial genomic DNA fragments that matched the genetic material in microbiota residing in the GIT [229]. An in vivo whole-body imaging study was performed to evaluate the absorption of bacteria and their EVs across the intestinal barrier and their movement into the host organs. *Pseudomonas panacis* cells and their EVs were orally administered to mice and the EVs were found to be present in the heart and lungs 5 min after their administration. Imaging data 12 h after the inoculation showed the systemic distribution of bEVs including organs, such as the liver, adipose tissue, and skeletal muscle [188]. These studies show that bEVs have the ability to cross the intestinal barrier and travel to distant organs and possibly release their cargo there.

*Helicobacter pylori*, a Gram-negative intestinal pathogen that colonizes the epithelial lining of the human stomach, has been listed as a Group 1 carcinogenic agent in humans by the International Agency for Research on Cancer (IARC) [110]. Though extensive studies have been carried out focusing on the role of *Helicobacter pylori* in the initiation and progression of gastric cancer, the role of *Helicobacter pylori*-derived bEVs in the pathogenesis of gastric cancer has largely remained unexplored. *Helicobacter pylori*-derived bEVs have been found to be upregulated in the gastric juice of gastric cancer patients when compared with normal controls [230]. Choi et al. (2017) [230] demonstrated that *Helicobacter pylori*-derived bEVs infiltrate into gastric mucosa as well as gastric epithelial cells. When *Helicobacter pylori*-derived bEVs labeled with Dil stain were applied to gastric epithelial cells and observed 12 h post administration, it was found that large numbers of vesicles were present inside the gastric epithelial cells [230]. CagA- and VacA-positive strains of *Helicobacter pylori* have already been associated with the induction of apoptosis in the adenocarcinoma gastric cell line (AGS) and the severity of the precancerous condition of gastric cancer [125]. The presence of CagA and VacA proteins has been demonstrated in EVs isolated from *Helicobacter pylori*. Turkina et al. (2015) [231] demonstrated that *Helicobacter Pylori* EVs containing CagA increase ATP affinity to H1 histone proteins in chromosomes, which may lead to the initiation of cancer [231,232]. A study conducted by Tyler et al. (2014) [217] showed the potential of bEVs isolated from *Escherichia coli* to induce carcinogenesis in intestinal epithelial cells. The internalization of *Escherichia coli*-derived bEVs into the Caco-2 cell line was determined by fluorescent dye labeling, and further investigations were carried out that showed an alteration of cell growth, damage to DNA, and aneuploidy in the presence of these bEVs [217]. These findings suggest that bEVs might infiltrate into gastric mucosa and gastric epithelial cells and possibly play an important role in GIT cancer.

Different studies have also indicated that bEVs could accumulate at the site of TME. Oh Youn Kim et al. (2017) [49] showed that intravenously injected *Escherichia coli* OMVs accumulated specifically near the tumor tissues in BALB/c mice bearing CT26 tumors. These bEVs attracted T cells and natural killer (NK) cells and induced the production of antitumor cytokines and interferons at the tumor site [49]. To avoid the possible adverse effects due to the bacterial endotoxin, bEVs were isolated from A acyltransferase (msbB) inactivated *Escherichia coli*. The bEVs were labeled with Cyanine7 (Cy7) fluorescence, and the fluorescence intensity of Cy7 in the whole body and different organs was measured with in vivo imaging system after 12 h of injection. Strong fluorescence signals were observed in tumor tissue. When the radiation efficiency of Cy7 was measured against each organ weight, the tumor tissue was found to have the highest intensity, suggesting accumulation of labeled bEVs in the tumor tissue site [49]. Likewise, Kudelaidi Kuerbana et al. (2000) [233] observed a massive accumulation of OMVs derived from attenuated *Klebsiella pneumonia* in the tumor area of BALB/c nude mice induced with a non-small-cell lung cancer (NSCLC) A549 cell line. When free doxorubicin hydrochloride (DOX) and an equivalent dose of DOX-loaded bEVs were introduced to A549 cell lines in vitro, a massive number of DOX-containing vesicles were observed in the tumor cells in 12 h post-infection in contrast to free DOX, which accumulated only after 24 h, indicating improved transport of the drug when it was encapsulated in the bEVs. It was also observed that DOX-OMVs could induce macrophages to release TNF-α and IL-6 [233]. When fluorescent-labeled bacterial vesicles of *Bacteroides thetaiotaomicron* were orally administered to C57BL/6 mice, high intensity signals were observed in the small intestine, stomach, caecum, and colon, while low intensity was observed in the liver, lungs, and heart after 8 h, indicating the bacterial vesicles could pass through the intestinal epithelial barrier and enter into the intestinal cells and even migrate to distant organs [228]. The same study indicated an uptake of bacterial vesicles within 48 h by the human colonic epithelial cell line Caco-2 when bacterial vesicles from *Bacteroides thetaiotaomicron* were incubated with the cell line [228]. When bEVs isolated from *Vibrio cholera*, *Escherichia coli* (BL21), and *Shigella flexneri* were intramurally injected into mice bearing a CT26 colon tumor, it was observed that all the membrane vesicles inhibited tumor growth significantly but in varying amounts, with the most pronounced inhibition caused by *Escherichia coli* (BL21) bEVs [77]. The naked bacterial vesicles resulted in inflammatory reactions due to the release of cytokines, such as interleukin-6 and tumor necrosis factor-α, that resulted in the death of the mice. Further studies were carried out only with vesicles isolated from *Escherichia coli* (BL21). The vesicles were labeled with Cy7 fluorescence and when injected into mice, showed accumulation of vesicles at the tumor site. But accumulation of vesicles decreased in subsequent injections. The membrane vesicles were encapsulated in biocompatible calcium phosphate by a biomineralization process to avoid adverse reactions and the experiment was repeated. Strong fluorescence signals were observed at tumor sites even after 24 h of final injection indicating efficient accumulation of the vesicles in the tumor site [77]. When global transcriptome profiling analysis was performed for tumors treated with membrane vesicles of *Escherichia coli*, the following were observed: significant gene suppression of immune suppressive genes such as *Tcaim* and *Socs6*, increased expression of immune activation-related genes such as *Tlrs* and *Gzmc*, and the upregulation of apoptosis genes *Nod1* and *Tnfrsf1a* [77].

A large number of studies have indicated the role of inflammation as a major risk factor in the promotion and progression of tumorigenesis including various GIT cancers such as CRC, gastric cancer, pancreatic cancers, and esophageal cancers [234,235]. The presence of inflammatory carcinogenic metabolites in cancer cells have been generally attributed to the interaction between the TME and resident microbiota [236,237]. bEVs have also been found to induce a significant influence on the expression of inflammatory mediators such as interleukins and nuclear factor kappa B (NF-κB) [238]. Since many compounds associated with inflammation are also found in bacterial membrane vesicles [239,240] and these bEVs are found near the TME, the possible role of bEVs in these processes cannot be ruled out.

Studies provide evidence that *Enterococcus faecalis* and *Escherichia coli* produce extracellular toxins causing chromosomal instability as well as free radicals targeting DNA, which can contribute to CRC development [225,241]. It is easy to speculate that the vesicles produced by these bacteria may also carry these toxins and free radicals and possibly cause the modulation of genetic material in host intestinal cells that could lead to oncogenesis. The microbial dysbiosis between healthy and cancerous conditions, along with the accumulation of bacterial vesicles in the TME, gives clues suggesting these vesicles released by the altered microbial community could act as tumor-promoting agents by invoking the immunogenic mechanism of the TME, via the suppression of immune cells, the production of carcinogenic metabolites, and the modification of the TME [49,77,242].

Although the direct involvement of bacterial pathogen in carcinogenesis has been reported [243,244], the involvement of tumor-influencing metabolic substances in a free state or packaged inside the bacterial membrane vesicle is a huge possibility [245,246]. Bacterial nucleic acid fragments have been detected in the host circulatory system [229], but it remains unclear whether these nucleic acids appeared due to the diffusion through the intestinal epithelium or were carried as cargo of the bacterial vesicles. The transfer of carcinogenic property might not be possible by free circulation of pathogenic bacteria or a bacterial component in host circulation, since these would elicit the host’s immune responses [247,248], leading to different host physiological changes. One possible explanation for this phenomenon may be the transfer of free metabolites or the cancer-inducing substances enclosed inside the bEVs, which can evade these host immune responses [20], and when presented with a favorable environment, induce carcinogenesis. Further investigation in this direction, to determine whether these vesicles carrying microbial nucleic materials are driving cancer pathogenesis or just coincidences, is essential.

Microbial dysbiosis in a cancerous condition, along with the accumulation of bacterial vesicles in the TME, gives indications that these vesicles released by the altered microbial community could act as tumor-promoting agents by invoking immunogenic mechanisms in the TME, via suppression of immune cells, production of carcinogenic metabolites, and modification of the TME [49,77,242]. Therefore, it is tempting to speculate that these vesicles might be one of the factors explaining the link between disturbances in the gut microbial community and oncogenesis.

All this evidence suggests that bEVs may not only have the capacity to enter the TME efficiently but might also influence the tumor environment by releasing various oncogenic metabolites or inducing the components of the TME to release them.

## 7. Interactions between bEVs and GIT Cancers

The last few decades have seen huge advancements in metagenomic, metabolomic, metaproteomic, and bioinformatics technologies in the field of biology [249,250,251,252]. These advancements in sequencing technologies and computational methods have enhanced the accurate and comprehensive analysis of microbial communities directly from the available source, without the complication of cultivation as reviewed in [249]. However, the use of these advanced technologies has been very limited in the analysis of bEVs related to GIT cancers. Along with the study of the process of biogenesis and the structure of the bacterial membrane vesicles, the content of the vesicles has also been a fascinating field of interest among scientists lately. Different biochemical, proteomic, and genetic analyses have shown that bEVs carry a large diversity of bioactive cargo compounds and an abundant number of metabolites as reviewed in [166,253,254,255].

### 7.1. Contents of bEVs That Potentially Affect GIT Cancers

#### 7.1.1. DNA

DNA fragments were found in *Pseudomonas aeruginosa* membrane vesicles when the bacteria lysed spontaneously, releasing membrane vesicles which contained cytosolic contents [174]. Many studies in the past have also reported DNA in the membrane vesicles of different bacteria such as *Neisseria gonorrhoeae*, *Streptococcus mutant*, and *Escherichia coli* [29,256]. The presence of DNA fragments in bacterial membrane vesicles has been attributed to the exchange of genetic material in the same and/or different species, facilitated by the membrane vesicles for the transfer of virulence factors and the antibiotic gene transfer between them [191,257]. Recent studies have shown that DNA cargo is carried into host cells inside membrane vesicles [62]. Studies have also indicated that bacterial DNA material integrated into the human genome more readily in tumor cells than in normal cells [60]. It remains undetermined whether the DNA carried within the vesicles may be the source of the integrated DNA [62]. The implications of vesicle-associated DNA fragments in host–pathogen interactions remains understudied.

#### 7.1.2. RNA

Recent studies have identified msRNAs and sRNAs in bEVs with sizes comparable to that of mRNA. The sRNA and msRNA have been thought to have regulatory functions in eukaryotes. Therefore, it has been speculated that these RNAs contained within bEVs participate in the modulation of the cancer microenvironment [258,259,260]. A high amount of mRNA encoding for DNA-binding proteins and membrane proteins along with DNA, tmRNA, and RNase P were also observed in the membrane vesicles of *Vibrio cholerae* O395 by Langlete et al. (2019) [261]. tRNA and rRNA fragments have been found in the membrane vesicles of *Escherichia coli* [254]. RNA profiling analysis of *Salmonella* spp.-derived membrane vesicles revealed higher RNA concentrations in EVs than in the cytosol [262].

#### 7.1.3. Protein

Liu et al. (2019) [263] recently performed proteomic analyses of *Fusobacterium nucleatum* EVs and found that 6 out of 98 proteins were autotransporter proteins [263]. One of these autotransporter proteins in *Fusobacterium nucleatum*, Fap2 protein, is known to provide protection to CRC cells by restraining the immune response, such as NK cell cytotoxicity, tumor-infiltrating lymphocytes, and a T-cell attack [264], by interacting with the T-cell immunoglobulin and ITIM (TIGIT) receptor domain [264,265] thus promoting tumor progression. An abundance of autotransporter proteins in *Fusobacterium nucleatum* EVs indicates the role of bEVs in cancer progression and opens up a whole new dimension for the study of microbial EV proteomics in cancer research.

#### 7.1.4. Metabolomes

In a metagenomic profiling study based on 16 S rDNA amplicon sequencing carried out on bEVs isolated from healthy volunteers and CRC patients demonstrated an alteration of gut microbiota with a significant increase in the abundance of Firmicutes and Proteobacteria in CRC subjects. In addition, *Proteus* spp. could only be detected in CRC patients. Similarly, metabolomic analysis carried out in the same study indicated phenol, ethanolamine, oxalic acid, succinic acid, furoic acid, palmitic acid, hexanoic acid, and oleic acid increased while butanoic acid was reduced in CRC patients. The authors summarized their findings by suggesting the combined use of metagenomic and metabolomic biomarkers for the diagnosis of CRC [241].

### 7.2. Potential Effects of bEVs on GIT Cancers

A study conducted by Vdovikova et al. (2018) [245] illustrated that bEVs from *Escherichia coli* and *Vibrio cholerae* were involved in the increase in gene expression associated with cellular differentiation in colon cancer cells. In their study, HCT8 cells from human ileocecal colorectal adenocarcinoma were co-cultured with membrane vesicles isolated from *Vibrio cholerae* and *Escherichia coli* for 5 h. RNA from membrane vesicle-treated cells were isolated and RNA sequencing performed. A total of 1434 and 685 genes were found to be differentially regulated by *Escherichia coli* membrane vesicles and *Vibrio cholerae* membrane vesicles, respectively. Approximately 51% (738 out of 1434) of the genes treated by *Escherichia coli* membrane vesicles and approximately 68% (465 out of 685) of the genes treated by *Vibrio cholerae* vesicles were significantly upregulated compared to control cells. The results suggest that *Vibrio cholerae* and *Escherichia coli* vesicles induce differential gene expression in HCT8 cells [245]. Similarly, to study the impact of membrane vesicles on the activation of gene transcripts in HCT8 cells, Vdovikova et al. (2018) [245] analyzed the distribution of reads from RNA sequencing experiments from transcription start sites (TSS) and the termination end sites (TES) at genes that were upregulated after vesicle treatment. They observed an increase in the H3K4me3 signal around the TSS of genes upregulated by both *Escherichia coli* and *Vibrio cholerae* membrane vesicles individually. The result showed membrane vesicles derived from both *Escherichia coli* and *Vibrio cholerae* impacted the activation of the gene transcription. The authors suggested that the study further enhances the current knowledge of the role of *Vibrio cholerae*-derived membrane vesicles on the differentiation of intestinal cancer via selective gene transcription [245]. This study helps to form a basis for further research on how bacterial vesicles influence gene expression of cells that might be influencing the cancer microenvironment.

An increased abundance of *Fusobacterium* spp. has been reported in the intestines of patients with CRC [77,266,267]. *Fusobacterium nucleatum* has been associated with the development, progression, and metastasis of CRC [266] by the stimulation of inflammatory pathways, such as the tumor necrosis factor (TNF) and nuclear factor-κB (NF-κB) pathways [267], increased production of CXCL1 and IL-8 [268] and drug resistance [269]. *Fusobacterium nucleatum* has also been found to activate Toll-like receptor 4 (TRL4) signaling to NF-κB, which resulted in an increased proliferation of CRC cells. A study conducted by Engevik et al. (2021) [50] indicated that OMVs secreted by *Fusobacterium nucleatum subsp. polymorphum* can activate TRL4 and influence the NF-κB pathway, thereby promoting proinflammatory cytokine production. When purified, OMVs from *Fusobacterium nucleatum* were applied to the human colon cell line HT-29, an approximately eight-fold increase in IL-8 production and an approximately six-fold increase in TNF production were observed [50].

All these studies indicate that there are a large number of genetic, proteomic, and metabolomic investigations that still need to be performed in order to expand our understanding of the interrelationship between cancer-associated bacterial vesicles and gastrointestinal cancer. Without a targeted study, one can only speculate about the possibility of interaction between tumor-associated bacteria with the host cells in the TME through bEV cargo content.

## 8. Clinical and Pharmaceutical Potential of bEVs

Oh Youn Kim et al. (2017) [49] demonstrated that when OMVs derived from A acyltransferase (*msbB*) inactivated *Escherichia coli* were intravenously injected into BALB/c mice bearing a CT26 colon tumor, these ∆*msbB* OMVs specifically targeted and accumulated in tumor tissue and fully eradicated established tumors by inducing the production of anti-cancerous cytokines CXCL10 and interferon-γ from NK and T cells. The team found a dose-dependent reduction in tumor volume with complete elimination of tumor tissue with 5 μg of ∆*msbB* OMVs. The mice were further challenged with CT26 colon tumor cells after 4 weeks and again after 3 weeks to study immunological memory; both of the challenges were rejected. Similar results were obtained when different strains of mice were subjected to MC38 colon cancer cells under similar conditions [49]. Similarly, Shuang et al. (2020) [77] reported that when OMVs prepared from *Escherichia coli* BL21 cells injected into mice bearing a CT26 tumor, they inhibited tumor growth to a significant level. Here, the OMVs were encapsulated with calcium phosphate (CaP) to prevent the inflammation that resulted when only OMVs were used. The encapsulation also enabled an efficient accumulation of OMVs at the tumor site even after repeated injections. At the tumor site, due to the acidic environment, the calcium phosphate shells dissolved, which helped to neutralize the acidic environment and thereby facilitated the immune cells to infiltrate, resulting in the promotion of antitumor responses [77]. The outer membrane of bEVs can be integrated with different biocompatible compounds [77,233] to prevent the adverse effects of naked vesicles and also increase the targeting potential of the vesicle. The integration of anti-tumor and chemotherapeutic drugs inside the protective layer of the vesicle along with enhanced tumor targeting and accumulation possess a enormous possibility for a future cancer treatment strategy. Similar studies using cell lines other than the CRC cell line have also been conducted and yield analogous results [233]. The outer membrane of bEVs may be integrated with different biocompatible compounds to make the vesicles more stable in the circulatory system. This experiment provides an insight into a novel method for the treatment of cancer.

Vesicles expressing pathogen-derived factors and changes in bEV abundance in host factors can serve as diagnostic biomarkers [270,271], indicators of disease progression, and capture of these pathogen-derived EVs may be used for further analysis. Changes in bEV composition during disease progression makes them excellent biomarker candidates [272]. The first blood-based EV diagnostic test for cancer became commercially available in January of 2016 in the United States [272,273]. If the disease-specific bEV biomarker becomes available, more chip-based diagnostic tools may be developed. Similarly, novel microbiome markers may be developed for the early diagnosis of GIT cancers by analyzing bEVs from blood samples.

It has been established that bEVs have the intrinsic targeting potential to selectively target other microorganisms as well as host cells [49,77]. In addition, different bioengineering techniques have been put forward to enhance the targeting properties of EVs [77]. If this cell-specific targeting potential could be augmented with the advanced imaging techniques for the labeling of the EVs in real-time imaging, a powerful diagnostic tool could be developed. The knowledge gained so far for tracking EVs may be used to develop novel, non-invasive imaging techniques, which can provide a better prospective of the in vivo therapeutic effects of bEVs by providing a precise glimpse of the in vivo distribution and dynamics of the EVs [210]. Efficient preclinical and clinical in vivo tracking techniques are key instruments for the development and optimization of vesicle-based diagnosis and treatment [274].

Since the bacterial vesicles are derived from different bacterial species with diverse membrane compositions and genetic makeups, these vesicles also have heterologous sizes, surface components, and diverse molecular cargo composition and, thus, have the capability for a variety of biological functioning as reviewed in [175]. The ability of these vesicles to cross the biological barrier, without causing adverse effects, remaining stable in the circulatory system and transporting its cargo specifically and selectively to the targeted TME may be utilized for various pharmaceutical purposes. Although it carries huge potential and possibility, the use of bEVs for treatment and as drug delivery vehicles in GIT cancer has been less studied.

Another advantage of bacterial vesicles is that the bacteria can be modified genetically to produce desired agents useful in imaging, therapy, and targeted delivery to be localized specifically in membrane-derived vesicles [275,276,277], and it may be mass-produced in large quantities [278,279]. With the ability to bioengineer vesicles to make them target-specific, ease of surface molecule modulation, imaging capabilities, mass production, and the ability to carry the desired payload, bEVs may emerge into a powerful theranostic tool [276,280,281].

Systemic administration of cancer drug-carrying bEVs may be a better alternative to oral administration due to the fact of their stability in the circulatory system, their ability to target and accumulate specifically at the tumor site, and their easy absorption into the cancer cells, thus reducing the higher dose and chemotoxicity of the cancer drugs. The outer membrane proteins can be modified to reduce endotoxicity by modifying the lipopolysaccharide pathways [282]. These vesicles can be loaded with the desired chemotherapeutic agents and anti-tumor agents, and outer proteins can be bioengineered to carry specific surface proteins to aid targeted therapy [49,283]. The nanosized and non-replicative status of EVs together with their resistance to enzymes and low pH, along with their ability to selectively interact with different types of mucosal and systemic host cells, makes them ideal candidates for drug delivery [284], targeted therapy, and imaging.

Adaptation of the studies carried out in cell lines and orthotopic modes into clinical translation presents another major hurdle. Further studies on this front are necessary, using different experimental designs so that the results of these findings could be translated to suit human patients.

Huge potential lies ahead in the utilization of bEVs for diagnosis, imaging, and targeted therapy for different kinds of cancers including cancers of the GIT.

## 9. Conclusions

There have only been a handful of studies dedicated to investigating the role of bEVs with respect to their impact on oncogenesis and tumor progression. In their review, Antonios Chronopoulos and Raghu Kalluri (2020) [20] suggested that bEVs might prove to be the important missing link between cancer and associated gut microorganisms [20]. An increased number of investigations using the latest technologies, such as metagenomics, proteomics, metabolomics, and bioinformatics, need to be carried out to understand the interaction between bEVs and GIT cancers. A bEV-based antitumor strategy could bring new insights for the development of novel cancer therapy in the future. These investigations will help to establish possible prevention, diagnosis, and treatment protocols for gastrointestinal cancer.

## Figures and Tables

**Table 1:** Global GIT Cancer Statistics 2020 [88,89].

Cancer	Incidence in 2020	Number of Deaths in 2020
Lip, oral cancer	377,713	177,757
Esophageal cancer	604,100	544,076
Stomach cancer	1,089,103	768,793
Colorectal cancer	1,931,590	935,173
Hepatic cancer	905,677	830,108
Pancreatic cancer	495,773	466,003

**Table 2:** Dysbiosis in GIT cancers.

Cancerous Condition	Normal Microflora in That Part of GIT	Increased in Cancer	Decreased in Cancer
Oral squamous cell carcinoma	*Streptococcus gordonii*, *Streptococcus mitis*, *Streptococcus sangius*, *Gemella sangius*, and *Granulicatella adiacens* [126] *Capnocytophaga*, *Fusobacterium*, *Lactobacterium*, *Porphyromonas*, *Peptostreptococcus*, *Staphylococcus*, Proteobacteria, and Actinobacteria [127]	*Streptococcus mitis*, and *Capnocytophaga* [101] *Fusobacterium*, *Dialister*, *Peptostreptococcus*, *Filifactor*, *Peptococcus*, *Catonella*, and *Parvimonas* [128]	Firmicutes and Actinobacteria [99] Streptococcaceae, Micrococcaceae Actinomycetaceae and Carnobacteriaceae, *Streptococcus*, *Veillonella*, and *Rothia* [103]
Esophageal adenocarcinoma	*Streptococcus viridians* [129] Firmicutes, Bacteroides, Actinobacteria, Proteobacteria, Fusobacteria, *Streptococcus* spp., *Haemophilus*, *Neisseria*, *Prevotella*, and *Veillonella*, [130,131]	Enterobacteriaceae, *Lactobacillus*, *Akkermansia*, and *Lactobacillus* [109,132]	Firmicutes *Veillonella*, and *Granulicatella* [109,132]
Esophageal squamous cell carcinoma		Proteobacteria, Bacteroidetes, Firmicutes and Spirochaetes [130,133] *Streptococcus*, *Prevotella*, *Porphyromona*, and *Treponema* [134,135,136]	*Lautropia*, *Bulleidia*, *Catonella*, *Corynebacterium*, *Moryella*, *Peptococcus*, and *Cardiobacterium* [135]
Gastric cancer	Firmicutes, Bacteroidetes, Actinobacteria, Fusobacteria, Proteobacteria, *Streptococcus*, and *Prevotella* [137,138,139,140]	Lachnospiraceae [111], *Achromobacter*, *Lactobacillus*, *Citrobacter*, *Clostridium*, and *Rhodococcus* [141], *Prevotella*, *Veillonella* [139] *Lactobacillus coleohominis*, *Klebsiella pneumoniae*, and *Acinetobacter baumannii* [111,112,140]	*Porphyromonas*, *Neisseria*, and *Streptococcus sinensis* [111,142]
Colorectal cancer	Bacteroides, Firmicutes [143], *Prevotella*, *Clostridium*, *Eubacterium* [144], *Lactobacillus*, *Streptococcus* [145], and *Acinobacter* [146]	*Fusobacterium*, *Porphyromonas*, *Peptostreptococcus*, and *Mogibacterium* Bacteroids [6,121,147] *Streptocpccus bovis*, *Helicobacter pylori*, *Escherichia coli*, *Enterococcus faecalis*, *Clostridium septicum*, and *Fusobacterium nucleatum* [117,118,148]	*Clostridium* and *Bacteroides*, *Pseudomonas*, *Prevotella*, *Acinetobacter*, and *Catenibacterium*, *Lactobacillus* and *Bifidobacterium*, *Rosebura*, and *Eupacteria* [121,147,148]
